# Microplastics Release From Clear Aligners: A Scoping Review of Current Evidence, Health Implications, and Policy Directions

**DOI:** 10.1155/ijod/6702035

**Published:** 2026-05-26

**Authors:** Christal Varghese, Shravan Shetty, M. V. Ashith, Madhumitha Natarajan, Radhika Agarwal, Athira Venugopal, Gulmehak Kalsi

**Affiliations:** ^1^ Department of Orthodontics and Dentofacial Orthopaedics, Manipal College of Dental Sciences Mangalore, Manipal Academy of Higher Education, Manipal, Karnataka, 576104, India, manipal.edu; ^2^ Department of Orthodontics and Dentofacial Orthopaedics, Manipal College of Dental Sciences, Manipal Academy of Higher Education, Manipal, Karnataka, 576104, India, manipal.edu

**Keywords:** clear orthodontic aligners, in-house aligners, microplastics, plastic pollution, polymers

## Abstract

**Background:**

Despite the dramatic increase in the use of orthodontic clear aligners in recent years, little is known about the presence of microplastics (MP) and their impact on human health. Through a scoping review study, we hope to map out the worldwide research on this intriguing topic of MP in orthodontic clear aligners.

**Methods:**

The eligibility of both published and unpublished articles was determined using a three‐stage search strategy suggested by the Joanna Briggs Institute. The databases PubMed, Scopus, Embase, and Web of Science were thoroughly searched. Only publications in English language were taken into consideration for the study, which included research done worldwide during the previous 10 years to February 2025. At any stage of the selection process, disagreements between the two reviewers over whether to include an article were settled by debate and consultation with a third reviewer. The gathered data was shown using a narrative summary and a PRISMA ScR checklist. This gave an overview of the most recent data about MP in clear aligners.

**Results:**

Literature search resulted in 638 articles out of which 250 duplicates were removed. Three hundred and eighty‐eight articles were screened for titles and abstracts out of which full text screening of 10 studies were done, and six articles proved the presence and the role of MP in Clear Aligners. Gray literature was also included in the scoping review.

**Conclusion:**

It can be concluded that although there is already evidence that transparent aligners release MP when in use, it is still unknown how much of a health danger they present. To solve these issues, more extensive in vivo research and the production of sustainable, safer materials and other policies are required.

## 1. Introduction

Over the past years, Clear Aligner Therapy (CAT) has become a revolutionary treatment option for traditional fixed orthodontic appliances, providing enhanced comfort, dental cleanliness, and aesthetics [1, 2]. This custom‐made, detachable orthodontic appliance made of transparent thermoplastic materials, are intended to gradually realign teeth [3, 4]. In contrast to braces, aligners can be removed for cleaning and eating, which improves periodontal health and lessens plaque buildup [5, 6].

Rubber tooth positioners were first introduced by Kesling in 1945 in an effort to improve orthodontic results [7, 8]. Later developments by Ponitz [9], McNamara [10], and Nahoum [11] improved vacuum‐formed, transparent plastic appliances [12–14]. Polyethylene terephthalate glycol (PETG), thermoplastic polyurethane (TPU) [15], and multilayer copolyesters are the most common materials used in aligners today. They are transparent, flexible, elastic, and biocompatible [14].

Although there are many therapeutic and patient‐centered advantages of CAT, questions have been raised about its potential effects on the environment and biology. MP, which are plastic particles smaller than 5 µm that might penetrate ecosystems and perhaps have an impact on human health, are produced because of the wear, thermoforming, and disposal of significant quantities of thermoplastic material [2]. The necessity for sustainable materials and waste management techniques in orthodontics is highlighted by the possibility of micro‐ and nano plastic fragments being released during intraoral disintegration brought on by mechanical stress, temperature fluctuations, and enzymatic activity [13]. Reviewing the present status of clear aligner materials, their clinical efficacy, and any possible effects on patients and the environment is pertinent given the quick development of aligner materials and production processes as well as the increased awareness of their environmental impact. This scoping review aims to gather and analyze available evidence on the presence and effects of MP from clear aligners, providing valuable insights into their biological impact. By highlighting current knowledge gaps, the review also emphasizes the need for further research to explore safer material alternatives and develop policies that minimize microplastic exposure to patients.

## 2. Methods

This scoping review was conducted using Arksey O’Malley’s framework [16], reported in accordance with PRISMA ScR guidelines [17] and the protocol was registered in OSF registries prior to commencement. This scoping review was conducted to answer the research question: “What is the current evidence on the release of MP from clear aligners, and what are the key gaps in research that need to be addressed for future studies?”

### 2.1. Information Sources

Literature search strategies were developed using medical subject headings (MeSH) and text words related to “Clear orthodontic aligners,” “in‐house aligners,” “Microplastics,” “Polymers,” and “Plastic pollution.” The databases considered for the search were PubMed, Scopus, Embase, and Web of Science. Gray literature was also considered. The search was conducted by one researcher (CR) with the last search conducted in February 2025.

### 2.2. Search Strategy

The search strategy included keywords related to orthodontic clear aligners, MP, and polymers. These search terms were adapted for use in PubMed, Scopus, Embase, and Web of Science databases. The search strategy used across the databases is given in Table 1.

**Table 1:** Search stratergy for PubMed, Embase, Scopus, Web of Science.

PubMed	(“Orthodontic appliances, removable”[MeSH Terms] OR ((“Clear”[All Fields] OR “cleared”[All Fields] OR “clearing”[All Fields] OR “clearings”[All Fields] OR “clears”[All Fields]) AND “orthodontic aligner”[Text Word]) OR “orthodontic appliances removable”[Text Word] OR “clear aligners”[Text Word] OR “Invisalign”[Text Word] OR “Aligners”[Text Word] OR “orthodontic aligners”[Text Word] OR “clear aligner treatment”[Text Word] OR (((“Clear”[All Fields] OR “cleared”[All Fields] OR “clearing”[All Fields] OR “clearings”[All Fields] OR “clears”[All Fields]) AND (“dental health services”[MeSH Terms] OR (“dental”[All Fields] AND “health”[All Fields] AND “services”[All Fields]) OR “dental health services”[All Fields] OR “dental”[All Fields] OR “dentally”[All Fields] OR “dentals”[All Fields])) AND “aligner”[Text Word]) OR (“in‐house”[All Fields] AND “Aligners”[Text Word])) AND (“Microplastics”[MeSH Terms] OR “Microplastics”[Text Word] OR “plastics”[Text Word] OR “polymers”[Text Word] OR “plastic pollution”[Text Word])
Embase	(‘Orthodontic appliances, removable’/exp OR ((Clear OR cleared OR clearing OR clearings OR clears) AND ’orthodontic aligner’) OR ’orthodontic appliances removable’ OR ’clear aligners’ OR Invisalign OR Aligners OR ’orthodontic aligners’ OR ’clear aligner treatment’ OR (((Clear OR cleared OR clearing OR clearings OR clears) AND (‘dental health services’/exp OR (dental AND health AND services) OR ’dental health services’ OR dental OR dentally OR dentals)) AND aligner) OR (in‐house AND Aligners)) AND (Microplastics/exp OR Microplastics OR plastics OR polymers OR ’plastic pollution’)
Scopus	(INDEXTERMS(“orthodontic appliances, removable”) OR ((ALL(Clear) OR ALL(cleared) OR ALL(clearing) OR ALL(clearings) OR ALL(clears)) AND TITLE‐ABS‐KEY(“orthodontic aligner”)) OR TITLE‐ABS‐KEY(“orthodontic appliances removable”) OR TITLE‐ABS‐KEY(“clear aligners”) OR TITLE‐ABS‐KEY(Invisalign) OR TITLE‐ABS‐KEY(Aligners) OR TITLE‐ABS‐KEY(“orthodontic aligners”) OR TITLE‐ABS‐KEY(“clear aligner treatment”) OR (((ALL(Clear) OR ALL(cleared) OR ALL(clearing) OR ALL(clearings) OR ALL(clears)) AND (INDEXTERMS(“dental health services”) OR (ALL(dental) AND ALL(health) AND ALL(services)) OR ALL(“dental health services”) OR ALL(dental) OR ALL(dentally) OR ALL(dentals))) AND TITLE‐ABS‐KEY(aligner)) OR (ALL(in‐house) AND TITLE‐ABS‐KEY(Aligners))) AND (INDEXTERMS(Microplastics) OR TITLE‐ABS‐KEY(Microplastics) OR TITLE‐ABS‐KEY(plastics) OR TITLE‐ABS‐KEY(polymers) OR TITLE‐ABS‐KEY(“plastic pollution”))
Web of Science	(“Orthodontic appliances, removable” OR ((Clear OR cleared OR clearing OR clearings OR clears) AND ”orthodontic aligner”) OR ”orthodontic appliances removable” OR ”clear aligners” OR Invisalign OR Aligners OR ”orthodontic aligners” OR ”clear aligner treatment” OR (((Clear OR cleared OR clearing OR clearings OR clears) AND (“dental health services” OR (dental AND health AND services) OR ”dental health services” OR dental OR dentally OR dentals)) AND aligner) OR (in‐house AND Aligners)) AND (Microplastics OR Microplastics OR plastics OR polymers OR ”plastic pollution”)

### 2.3. Eligibility Criteria

In this scoping review, the inclusion and exclusion criteria were based on the Population, Concept, and Context framework.

#### 2.3.1. Population

Research papers and experimental studies that will shed light on the role of MP in clear aligners, which make up the population component of the PCC paradigm, will be included in this scoping review. These studies examine several topics, such as the methods by which mechanical wear releases MP, the possible health risks associated with ingestion, and the characteristics of the materials that affect deterioration.

#### 2.3.2. Concept

This scoping review focuses on MP as the central concept, specifically their presence, release, and impact in the context of clear aligners.

#### 2.3.3. Context

This scoping review’s global context includes studies conducted in different parts of the world to give a thorough grasp of microplastic release in clear aligners. The issue of microplastic production and its possible health and environmental ramifications is of global importance due to the widespread usage of transparent aligners in orthodontic treatments among many populations.

Original research articles published between February 2015 and February 2025 in English with full text availability were considered. Review articles, letters to editors, editorials, and articles were also included.

Articles that addressed plastic pollution in a broad sense without specifically addressing MP in clear aligners were not included in the review. Six hundred and thirty‐eight articles were found in the first search, duplicates were eliminated, and the remaining articles were filtered using the predetermined inclusion. Only those who satisfied the study’s goals were kept for additional assessment.

### 2.4. Study Selection

The search results obtained were managed using Mendeley software version 2.77.0 Mendeley Ltd. [18]. Initial title screening was performed in the Rayyan software by one author (CR), which was followed by title and abstract screening and full‐text review by the second, third, and fourth authors (SS, AM, MN). Any disagreements in study selection were resolved by discussion with the fifth, sixth, and seventh authors (RA, AV, GK), and consensus was achieved.

### 2.5. Data Extraction

Data‐charting form was jointly developed by seven reviewers (CV, SS, AM, MN, RA, AV, GK) to determine which variables to extract. The five reviewers independently charted the data, discussed the results, and continuously updated the data‐charting form in an iterative process. Any disagreements were discussed with the sixth and seventh reviewers (RA and GK). A review of the full text and any doubts or disagreement were discussed among all the authors and settled.

### 2.6. Synthesis of Results

This process included multiple readings of the selected articles to identify significant text segments, which were initially coded independently by two researchers (CV and SS). Subsequently, these initial codes and the full‐text articles were revisited to refine codes and establish main themes through discussions involving a third and fourth researchers (AV and RA).

Although an optional step in scoping review methodology, quality appraisal was undertaken to systematically identify existing research gaps, critically evaluate the methodological rigor of current studies, and guide future research directions in this field. The Crowe Critical Appraisal Tool (CCAT) was employed to assess the quality of the sources of evidence for the present scoping review [18]. Table 2 shows the Crowe’s critical appraisal scores of the included studies. CCAT involves evaluating each article across eight categories, with scores ranging from 0 to 5 for each category and a maximum score of 40 for each article. Based on the CCAT scores, the articles were scored as high quality with scores more than 35, medium quality with scores between 25 and 34, and low quality for scores below 25. CCAT is limited to original research studies, as other included sources lack methodological frameworks suitable for structured appraisal.

**Table 2:** Crowe’s critical appraisal scores of the included studies [18].

Author and year of publication	Preliminaries	Introduction	Design	Sampling	Data collection	Ethical	Results	Discussion	Total	Quality of study
Quinzi et al. (2023) [19]	4	4	4	3	4	4	4	4	31	Medium
Barlie et al. (2025) [20]	5	4	5	5	5	5	4	5	38	High

## 3. Results

**Figure 1:**
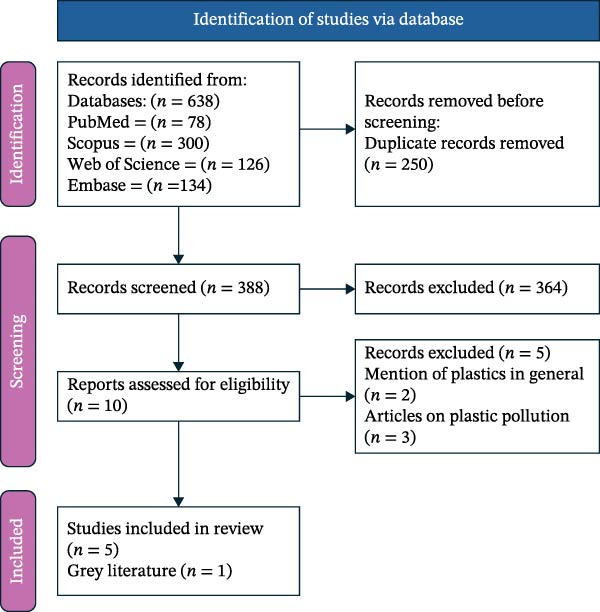
PRISMA flow diagram for scoping review results.

Following the database search, a total of 638 articles were reviewed. After elimination of 250 duplicates, a total of 388 articles were obtained. After title and abstract screening, 378 articles were removed, and full text review of 10 articles was done, and finally six articles were considered for the study out of which 1 gray literature was also included. The PRISMA flow diagram is given in Figure 1. The data obtained were summarized under the following headings: author name, date of publication, type of study, aim, results, limitations, and challenges. The study comprised in vitro studies, material analyses, and narrative reports. No clinical or longitudinal human studies were identified.

The characteristics of the selected studies were extracted by two independent researchers (CV and SS) into a predecided format. The extraction tables were compared and combined to fill in any missing information wherever applicable. The final data extraction table is provided in Table 3.

**Table 3:** Data extraction chart.

Serial no.	Author, date of publication	Reference no.	Type of study	Aim	Results	Limitations and challenges
1.	Quinzi et al., Jan 2023	[19]	In vitro experimental	To evaluate microplastic detachment from clear aligners in artificial saliva	MPs released from all aligners; polyurethane‐based (INV) released the fewest; size range mostly 5–20 µm	In vitro setup may not fully replicate complex oral environment (e.g., enzymatic activity, mastication). Limited to 7‐day exposure.
2.	Barile. et al., Feb 2025	[20]	Mechanical fatigue simulation	To study damage/MP release under cyclic loading simulating swallowing	MPs were released from all; Ghost Aligner showed poor mechanical durability; particle sizes not deemed health‐threatening	Aligner material data for Invisalign not disclosed; limited sample diversity; only 3 aligner brands tested.
3.	Panayi et al., Nov 2024	[21]	Review article	To summarize MP release and health risks from aligners	MPs may affect multiple organs and systems (brain, heart, gut); BPA leaching adds toxicity concern	Lacks new experimental data; relies on previous literature; does not quantify exposure or dose‐response.
4.	Vinod Krishnan, June 2024	[22]	Editorial summary	Raise awareness on risks of MPs from orthodontics	MPs >20 µm mostly excreted, but 5–20 µm may enter bloodstream and cause inflammation	Largely opinion‐based; not peer‐reviewed research; lacks direct experimental backing.
5.	Balasundaram et al., Oct 2023	[23]	Narrative review	To highlight dental sources of MPs and their risks	MPs and NPs released via mechanical degradation; risk of systemic oxidative and inflammatory effects	Lacks experimental verification; overview is general and non‐quantitative; based on secondary sources.
6.	Venugopal, May 2024	[24]	Letter to the editor	Alert dental professionals about potential MNP risks	MNPs may reach cardiovascular system; long‐term health impact needs investigation	Letter format restricts detail; no experimental data; raises concern without proposed solutions.

Clear orthodontic aligners, which are made of thermoplastic materials like polyethylene terephthalate (PET) and polyurethane (PU), frequently release secondary MP into the oral cavity [22]. Some of the reasons for this separation include intraoral aging processes such as water absorption and proteinaceous biofilm apposition, cyclic loading that simulates swallowing motions, and mechanical friction during daily wear in artificial saliva [20]. Research indicates that 3D‐printed aligners tend to release fewer and smaller MPs than thermoformed ones, such as Essix Ace and Ghost Aligner, because to better dimensional repeatability and less mechanical friction [25]. A number of these MPs may be smaller than 5 µm, but the majority are typically between 5 and 20 µm or greater than 20 µm, making their size crucial for evaluating any health hazards [22]. MPs between 5 and 20 µm may cross the gastrointestinal epithelium and perhaps enter the circulatory system by endocytosis or paracellular diffusion, while MPs bigger than 20 µm are likely to be eliminated from the gastrointestinal tract. Cell membranes can be passively crossed by particles as tiny as 1 µm or less than 5 µm [25]. With wider systemic effects on respiratory, cardiovascular, and neurological health, ingested MPs can cause oxidative stress, inflammatory lesions, chronic inflammation, change of gut microbiota, and an elevated risk of neoplasia [2, 26].

While the comfort and attractiveness of clear aligners have revolutionized orthodontic treatment, mounting research indicates that they can also release MP while in use. These particles, which are produced by aging, friction, and industrial processes, can be harmful to human health. The release of MP highlights the need for more in vivo study to elucidate the biological impact of aligners, even if they are still a useful therapy option. To reconcile therapeutic efficacy with patient safety and environmental responsibility, future developments must concentrate on creating safer, more sustainable materials and production techniques.

## 4. Discussion

Clear aligners have faced a rapid surge in demand over the past few years. This can be attributed to the increasing demand for aesthetic and a more comfortable treatment as compared to conventional fixed orthodontic appliances. As there is an increase in the demand for this aesthetic treatment modalities, there is also immense research on the improvement in aligner materials and new innovations for its faster and easier production. Depending on the complexity of the situation, the length of treatment can range from a few months to more than a year. Aligners are typically worn for 20–22 h every day and replaced every 1–2 weeks [7]. The advantages of clear aligners over conventional treatment include better oral hygiene, lesser appointments with lesser duration, fewer emergency visits, lesser pain on tooth movement. However, it also has a few disadvantages which include more production cost, patient compatibility is a must, and it has certain limitations in treatment of complex malocclusions [23]. Although the discrete look, comfort, and convenience of transparent aligners have revolutionized orthodontic treatment, there is a hidden danger that requires more investigation.

New research is beginning to highlight possible dangers related to long‐term usage. One such issue is the release of chemicals and MP from the aligner material, especially when it is exposed to heat, wear, and other chemicals in the oral environment. Unknown biological consequences might result from these compounds, particularly after prolonged contact. MPs are a significant concern in various fields, and their presence and detachment from clear orthodontic aligners have become a growing area of scientific investigation [22]. MP are generally defined as small plastic particles or fibers ranging from 1 to 5000 μm in diameter [25]. However, the lower size limit has been extended down to 100 nm by the European Food and Safety Authority (EFSA) [27]. MPs are categorized into two main types: Primary and secondary MP. Primary MP are intentionally manufactured as microbeads, capsules, fibers, or pellets and are found in products like toothpaste, face wash, cosmetics, and industrial abrasives [20] and secondary MP arise from the physical, chemical, and/or biological fragmentation and degradation of larger plastic objects during their use or when released into the environment [21]. Clear aligners are primarily made from thermoplastic materials such as PET, polypropylene (PP), polycarbonate (PC), and PU [28]. These materials are susceptible to degradation from various environmental and mechanical factors. The primary mechanism for MP detachment from clear aligners in the oral cavity is mechanical friction, which is the physiological friction generated by teeth [25]. This continuous frictional contact occurs between the occlusal aligner surfaces during daily wear and swallowing [22]. Quinzi et al. [19] in his study has shown that even a seven‐day wear protocol in artificial saliva can lead to the release of secondary MP due to this mechanical friction. The process of “aging” in aligners, exacerbated by the harsh oral cavity environment (pH and temperature fluctuations, saliva, enzymes, biting force), also contributes to the increase in roughness, friction, attrition, and wear, leading to MP detachment [29].

The manufacturing process of aligners also plays a significant role in MP detachment. Many aligners are produced by thermoforming plastic discs over 3D‐printed models [28]. This process involves heat generation, which can alter the material properties and mechanical characteristics, potentially leading to easier detachment of MPs [28]. Direct 3D‐printed aligners are manufactured using 3D printing technologies (stereolithography). This method can minimize material loss during production and may result in fewer MPs being detached. For instance, Invisalign aligners showed the lowest number of detached MPs in one study [22]. Some detected MPs were found to be pigmented with blue and black colors, likely from the ink used to identify the aligners. These areas may be less resistant to mechanical friction, leading to easier detachment of MPs [22]. Heat is applied during the manufacturing of thermoformed aligners, such as Ghost Aligner and Essix Ace, which can drastically alter their material characteristics. Thermal stress may be introduced during this process, resulting in localized stress zones that eventually induce aligner failure and MP separation by spreading as microcracks. In contrast, direct 3D‐printed aligners have higher dimensional repeatability, which might lead to reduced mechanical friction during operation and, in turn, less MP detachment. Eliades and Eliades [30] in their article have suggested that both aligners and composite attachments may contribute to microplastic release, as frictional interactions and wear between these materials can lead to the generation of particulate matter in the oral cavity.

Techniques like Raman micro spectroscopy, Scanning Electron Microscopy (SEM), Optical Microscopy and Image Segmentation, and Dimensional Analysis can be used for identification and characterization of MP. Raman Micro spectroscopy (RMS) is a highly reliable technique for detecting and identifying MPs, allowing the characterization of both morphological features and chemical composition. It can analyze MPs as small as ~2 μm directly on filtration membranes [31]. SEM is crucial for quantifying MPs and obtaining high‐resolution images of their micromorphology, including size, shape, and surface structure [22]. Optical microscopy is used for visual inspection and morphological characterization of detected MPs [25].

Quinzi et al. in their article shed light on the shape of MP in different aligner brands. The irregular MPs from Essix Ace ranged in size from 685 to 5097 µm^2^ [19]. Their minor axis lengths ranged from 14 to 64 µm, whereas their major axis lengths usually ranged from 30 to 65 µm, with some surpassing 111 µm whereas, the MPs from Ghost Aligner ranged from around 180 to 4400 µm^2^. Notably, at least one major axis of numerous pieces was less than 1 µm. Following testing, many microcracks were found in Ghost Aligner samples, which are in line with its subpar mechanical test results. The MPs obtained from Invisalign typically had the lowest average areas, ranging from 150 to 440 µm^2^. Their minor axis diameters ranged from 6 to 10 µm, while their major axis dimensions were approximately between 12 and 23 µm. Minimal damage zones were seen on the aligners, and only few MPs were retrieved from Invisalign.

The rapid global adoption of CAT has led to a significant rise in plastic waste generation. From manufacturing inefficiencies to disposable accessories, the treatment process produces thousands of tons of discarded plastic annually. This growing environmental burden underscores the urgent need for sustainable production and waste management strategies in orthodontics. In 2020, 23 years after the first release of Invisalign, Align Technology, a prominent maker of aligners, teamed up with TerraCycle [32] to provide recycling alternatives. Align Technology makes promises to enhance the use of renewable energy, decrease waste and emissions, recycle plastics, and minimize product packaging. It is unclear, meanwhile, how successful their experimental recycling efforts will be in the long run and whether the aligners that are collected are recycled into new items or are just disposed of in different ways.

Waste management adheres to the 4Rs: reduce, reuse, recycle, and recover [32]. Modern polymers may be used to reduce material usage, production can be optimized, and direct 3D printing can be used to reduce losses in clear aligner waste. Innovations like shape‐shifting 4D aligners may reduce the number of aligners required, even though reuse is presently unfeasible. Although theoretically feasible, recycling is still not widely used, and recovery techniques like pyrolysis or gasification provide substitute disposal choices [32].

Human health may also be seriously threatened by MP and nano plastics (NPs), mainly because of their widespread distribution and variety of entry points into the body [26]. The primary route of exposure of aligner MP is through ingestion. Both the physical characteristics of MP and the chemical additives they contain or absorb are responsible for their toxicity to humans [33]. MPs can physically result in inflammation, organ obstruction, and cell damage. Because they are thought to be able to pass across cell membranes, the blood‐brain barrier, and the placental barrier. NPs (less than 1 µm) are of particular concern because they can reach nearly every organ and fetal tissue. Numerous additives that leak from plastics and are referred to as endocrine disruptors, carcinogens, or neurotoxins—including heavy metals, bisphenol A (BPA), phthalates, and polychlorinated biphenyls (PCBs)—cause chemical toxicity [34]. Hormone disturbance, cardiovascular disease, cancer, developmental and reproductive toxicities, cognitive deficiencies, and immune system dysfunctions are just a few of the health problems that can result from exposure to these compounds. They are known to affect the digestive, respiratory, immune, and reproductive systems [32]. Abbas et al. in their article explain how microplastics (MP) like polyethylene, polypropylene, and polystyrene are now widely present in the air, water, and food. They enter the body through ingestion and inhalation and may cause gastrointestinal inflammation, immunological dysregulation, respiratory damage, cardiovascular risks, neurotoxicity, endocrine disruption, and possible reproductive disorders [35]. Both nano‐plastics and MP bioaccumulate in the lungs, placenta, blood, and digestive tract. There, they may cause cytotoxic and genotoxic effects, disrupt gut microbiota, and be linked to ailments like asthma, inflammatory bowel disease, and neurological symptoms. Reported systemic effects of MP, such as inflammatory responses, oxidative stress, and potential tissue‐level interactions, are primarily derived from environmental and experimental studies; however, the extent to which MP released from clear aligners contribute to these outcomes remains unclear and requires further targeted research.

Studies clearly show that there is microplastic release from clear aligners and these MP when they enter into the body may cause various adverse effects. However further research and in vivo experiments must be conducted to study how the release of MP can be reduced and the true degree of release of these particles from clear aligners. Studies must be done to develop better alternatives and policies to the conventional clear aligner materials which will thereby reduce the number of MP released and provide safe treatment for clear aligner patients. Research should be undertaken to find out policies to address the disposal and waste management of the aligners after their use.

## 5. Conclusion

This scoping review underlines a vital need for additional solid, long‐term epidemiological research to properly understand the association between microplastic exposure and unfavorable effects it has on different aspects of human life. Although the data now available supports their existence, it is nevertheless constrained by small sample numbers, brief observation times, and the lack of standardized testing procedures. There is an urgent need for thorough, long‐term research to measure microplastic release under actual oral conditions, evaluate related biological impacts, and investigate safer, biodegradable materials in addition to introducing policies that ensure sustainable manufacturing and recycling solutions, given the potential for systemic exposure and unknown long‐term effects.

**Nomenclature**
MP: Microplastics
OSF: Open science framework
CAT: Clear aligner therapy
CAD/CAM: Computer aided designing/computer aided machining
PETG: Polyethylene terephthalate glycol
TPU: Thermoplastic polyurethane
BPA: Bisphenol A
PET: Polyethylene terephthalate
PP: Polypropylene
PC: Polycarbonate
PU: Polyurethanes
SEM: Scanning electron microscopy
RMS: Raman micro spectroscopy
PCB: Polychlorinated biphenyls
EFSA: European Food and Safety Authority
NP: Nano plastics.

## Author Contributions

Christal Varghese, Shravan Shetty, M. V. Ashith, Radhika Agarwal, Gulmehak Kalsi, and Athira Venugopal contributed to the conceptualization of this review. Christal Varghese and Shravan Shetty contributed to the acquisition, analysis, and interpretation of data and drafted the manuscript. Athira Venugopal, M. V. Ashith, and Shravan Shetty critically revised the manuscript. Shravan Shetty provided supervision and mentorship.

## Funding

The authors have nothing to report.

## Ethics Statement

The authors have nothing to report.

## Conflicts of Interest

The authors declare no conflicts of interest.

## Data Availability

The authors have nothing to report.

## References

[1] Cenzato N. , Di Iasio G. , Martìn Carreras-Presas C. , Caprioglio A. , and Del Fabbro M. , Materials for Clear Aligners—A Comprehensive Exploration of Characteristics and Innovations: A Scoping Review, Journal of Clinical Orthodontics: JCO. (2024) 14, no. 15, 10.3390/app14156533, 6533.

[2] Hartogsohn C. R. and Sonnesen L. , Clear Aligner Treatment: Indications, Advantages, and Adverse Effects—A Systematic Review, Dentistry Journal. (2025) 13, no. 1, 10.3390/dj13010040. PMC11764167 39851616

[3] Girish Kumar I. , Clear Aligner Therapy: A Paradigm Shift in Orthodontics Review Article, Asian Journal of Dental Sciences. (2024) 7, 32–40.

[4] Gold B. P. , Siva S. , Duraisamy S. , Idaayath A. , and Kannan R. , Properties of Orthodontic Clear Aligner Materials-A Review, Journal of Evolution of Medical and Dental Sciences. (2021) 10, no. 37, 3288–3294, 10.14260/jemds/2021/668.

[5] Macrì M. , Murmura G. , Varvara G. , Traini T. , and Festa F. , Clinical Performances and Biological Features of Clear Aligners Materials in Orthodontics, Frontiers in Materials. (2022) 9, 819121.

[6] Monisha J. and Peter E. , Performance of Clear Aligners: Is It Overrated?, American Journal of Orthodontics and Dentofacial Orthopedics. (2023) 163, no. 6, 10.1016/j.ajodo.2023.03.001. 37245889

[7] Upadhyay M. and Arqub S. A. , Biomechanics of Clear Aligners: Hidden Truths & First Principles, Journal of the World Federation of Orthodontists. (2022) 11, 12–21. 34965910 10.1016/j.ejwf.2021.11.002

[8] Gierie W. V. , Clear Aligner Therapy: An Overview, Journal of Clinical Orthodontics. (2018) 52, no. 12, 665–674. 30576290

[9] Ponitz R. J. , Invisible Retainers, American Association of Orthodontists. (1971) 59, no. 3, 266–272, 10.1016/0002-9416(71)90099-6, 2-s2.0-0015028033. 5276727

[10] McNamara J. A. , Kramer K. L. , and Juenker J. P. , Invisible Retainers, Journal of Clinical Orthodonics. (1985) 19, no. 8, 570–578. 3862671

[11] Nahoum H. , The Vacuum Formed Dental Contour Appliance, The New York State Dental Journal. (1964) 9, 385–390.

[12] Kundal S. and Shokeen T. , Aligners: The Science of Clear Orthodontics, International Journal of Dental and Medical Specialty. (2020) 7, no. 1, 38–42.

[13] Bichu Y. M. , Alwafi A. , and Liu X. , et al.Advances in Orthodontic Clear Aligner Materials, Bioactive Materials. (2023) 22, 384–403, 10.1016/j.bioactmat.2022.10.006. 36311049 PMC9588987

[14] Lo I. L. , Kao C. Y. , Huang T. H. , Te Ho C. , and Kao C. T. , The Cytotoxicity Assessment of Different Clear Aligner Materials, Journal of Dental Science. (2024) 19, no. 4, 2065–2073, 10.1016/j.jds.2024.05.025. PMC11437316 39347034

[15] Arksey H. and O’Malley L. , Scoping Studies: Towards a Methodological Framework, International Journal of Social Research Methodology: Theory and Practice. (2005) 8, no. 1, 19–32, 10.1080/1364557032000119616, 2-s2.0-14644388070.

[16] Tricco A. C. , Lillie E. , and Zarin W. , et al.A Scoping Review on the Conduct and Reporting of Scoping Reviews, BMC Medical Research Methodology. (2016) 16. 10.1186/s12874-016-0116-4 PMC4746911 26857112

[17] Download Mendeley Reference Manager For Desktop Windows , Mendeley [Internet], 2025, https://www.mendeley.com/download-reference-manager/windows.

[18] Crowe M. , Sheppard L. , and Campbell A. , Reliability Analysis for a Proposed Critical Appraisal Tool Demonstrated Value for Diverse Research Designs, Journal of Clinical Epidemiology. (2012) 65, no. 4, 375–383, 10.1016/j.jclinepi.2011.08.006, 2-s2.0-81855225077. 22078576

[19] Quinzi V. , Orilisi G. , Vitiello F. , Notarstefano V. , Marzo G. , and Orsini G. , A Spectroscopic Study on Orthodontic Aligners: First Evidence of Secondary Microplastic Detachment after 7 Days of Artificial Saliva Exposure, Science of the Total Environment. (2023) 866, 10.1016/j.scitotenv.2022.161356, 161356. 36603638

[20] Barile C. , Cianci C. , and Paramsamy Kannan V. , et al.Experimental Assessment of Damage and Microplastic Release During Cyclic Loading of Clear Aligners, PLoS One. (2025) 20, no. 2, e0318207. 39908302 10.1371/journal.pone.0318207 PMC11798498

[21] Panayi N. , Papageorgiou S. N. , Eliades G. , and Eliades T. , Microplastics and Orthodontic Aligners: The Concerns Arising From the Modernization of Practice Through Polymers and Plastics, Journal of the World Federation of Orthodontists. (2024) 13, 259–264. 39567342 10.1016/j.ejwf.2024.10.001

[22] Krishnan V. , Microplastics: An Orthodontic Concern!, Journal of the World Federation of Orthodontists. (2024) 13, 103–104. 38830718 10.1016/j.ejwf.2024.05.001

[23] Balasundaram N. , Anjusha C. K. , Santhosh Kumar V. , and Nivetha R. , Microplastics in Orthodontics, International Journal of Contemporary Dental Research. (2023) 1, no. 4, 18–20, 10.62175/apdch2321.

[24] Rao S. , Shenoy R. , and Rao A. , et al.Exploring the Impact and Implications of Oral Health Interventions Among Older Adults: A Scoping Review, Journal of International Oral Health. (2025) 17, no. 4, 266–275, 10.4103/jioh.jioh_71_25.

[25] Lalrinfela P. , Vanlalsangi R. , Lalrinzuali K. , and Babu P. J. , Microplastics: Their Effects on the Environment, Human Health, and Plant Ecosystems, Environmental Pollution and Management. (2024) 1, 248–259, 10.1016/j.epm.2024.11.004.

[26] Tamer I. , Öztas E. , and Marsan G. , Orthodontic Treatment With Clear Aligners and the Scientific Reality Behind Their Marketing: A Literature Review, Turkish Journal of Orthodontics. (2019) 32, no. 4, 241–246, 10.5152/TurkJOrthod.2019.18083. 32110470 PMC7018497

[27] Macrì M. , D’Albis V. , Marciani R. , Nardella M. , and Festa F. , Towards Sustainable Orthodontics: Environmental Implications and Strategies for Clear Aligner Therapy, Materials. (2024) 17, 4171. 39274561 10.3390/ma17174171 PMC11395928

[28] Tamargo A. , Molinero N. , and Reinosa J. J. , et al.PET Microplastics Affect Human Gut Microbiota Communities During Simulated Gastrointestinal Digestion, First Evidence of Plausible Polymer Biodegradation During Human Digestion, Scientific Reports. (2022) 12, no. 1. 10.1038/s41598-021-04489-w PMC8752627 35017590

[29] Camcı H. and Büyükbayraktar Z.Ç. , Aligners From Another Perspective: Could They be a Long-Term Environmental Threat? Problems and Potential Remedies, American Journal of Orthodontics and Dentofacial Orthopedics. (2025) 167, no. 3, 256–260, 10.1016/j.ajodo.2024.10.016. 39641708

[30] Eliades T. and Eliades G. , Intraoral Ageing of Aligners and Attachments: Adverse Effects on Clinical Efficiency and Release of Biologically-Active Compounds, Korean Journal of Orthodontics. (2024) 54, no. 4, 199–209, 10.4041/kjod24.085. 38926752 PMC11270147

[31] Yuan Z. , Nag R. , and Cummins E. , Human Health Concerns regarding Microplastics in the Aquatic Environment-From Marine to Food Systems, Science of the Total Environment. (2022) 823, 153730. 35143789 10.1016/j.scitotenv.2022.153730

[32] Winiarska E. , Jutel M. , and Zemelka-Wiacek M. , The Potential Impact of Nano- and Microplastics on Human Health: Understanding Human Health Risks, Environmental Research. (2024) 251, 118535. 38460665 10.1016/j.envres.2024.118535

[33] Campanale C. , Massarelli C. , Savino I. , Locaputo V. , and Uricchio V. F. , A Detailed Review Study on Potential Effects of Microplastics and Additives of Concern on Human Health, International Journal of Environmental Research and Public Health. (2020) 17, no. 4, 10.3390/ijerph17041212, 1212. 32069998 PMC7068600

[34] Abbas G. , Ahmed U. , and Ahmad M. A. , Impact of Microplastics on Human Health: Risks, Diseases, and Affected Body Systems, Microplastics. (2025) 4.

[35] Zhu X. , Wang C. , Duan X. , Liang B. , Genbo Xu E. , and Huang Z. , Micro- and Nanoplastics: A New Cardiovascular Risk Factor?, Environment International. (2023) 171, 107662. 36473237 10.1016/j.envint.2022.107662

